# ER Stress-Activated HSF1 Governs Cancer Cell Resistance to USP7 Inhibitor-Based Chemotherapy through the PERK Pathway

**DOI:** 10.3390/ijms25052768

**Published:** 2024-02-27

**Authors:** Chang-Hoon Lim, Xue-Quan Fang, Hyeji Kang, Taerim Oh, Seonghoon Lee, Young-Seon Kim, Ji-Hong Lim

**Affiliations:** 1 Department of Medicinal Biosciences, College of Biomedical & Health Science, Konkuk University, 268, Chungwon-daero, Chungju 27478, Chungbuk, Republic of Korea; lchoo1196@kku.ac.kr (C.-H.L.); gkrrnjs654852@kku.ac.kr (X.-Q.F.); kkang@kku.ac.kr (H.K.); dhxofla555@kku.ac.kr (T.O.); samron7@kku.ac.kr (S.L.); yskim0801@kku.ac.kr (Y.-S.K.); 2 BK21 Program, Department of Applied Life Science, Graduate School, Konkuk University, 268, Chungwon-daero, Chungju 27478, Chungbuk, Republic of Korea; 3 Center for Metabolic Diseases, Konkuk University, 268, Chungwon-daero, Chungju 27478, Chungbuk, Republic of Korea

**Keywords:** chemoresistance, ubiquitin-specific protease 7 inhibitor, heat shock transcription factor 1, endoplasmic reticulum stress

## Abstract

Ubiquitin-specific protease 7 inhibitors (USP7i) are considered a novel class of anticancer drugs. Cancer cells occasionally become insensitive to anticancer drugs, known as chemoresistance, by acquiring multidrug resistance, resulting in poor clinical outcomes in patients with cancer. However, the chemoresistance of cancer cells to USP7i (P22077 and P5091) and mechanisms to overcome it have not yet been investigated. In the present study, we generated human cancer cells with acquired resistance to USP7i-induced cell death. Gene expression profiling showed that heat stress response (HSR)- and unfolded protein response (UPR)-related genes were largely upregulated in USP7i-resistant cancer cells. Biochemical studies showed that USP7i induced the phosphorylation and activation of heat shock transcription factor 1 (HSF1), mediated by the endoplasmic reticulum (ER) stress protein kinase R-like ER kinase (PERK) signaling pathway. Inhibition of HSF1 and PERK significantly sensitized cancer cells to USP7i-induced cytotoxicity. Our study demonstrated that the ER stress–PERK axis is responsible for chemoresistance to USP7i, and inhibiting PERK is a potential strategy for improving the anticancer efficacy of USP7i.

## 1. Introduction

The ubiquitin-specific protease 7 (USP7, also known as HAUSP) is widely recognized as a critical molecule participating in cancer development, growth, chemoresistance, and metastasis [1]. USP7 is known to be occasionally overexpressed in several cancers, and this overexpression has been reported to be closely associated with the chemoresistance of tumors to anticancer chemical drugs and increased metastatic ability, resulting in poor clinical outcomes in patients with cancer [2,3,4]. Over the last decade, multiple classes of USP7 inhibitors (USP7i) have been discovered, and their anticancer efficacies have been evaluated using in vitro and in vivo models [5,6]. Despite the current lack of relevant inhibitors entering clinical trials, USP7i are considered promising chemical drugs for cancer treatment.

Multidrug resistance to anticancer drugs, also called chemoresistance, is a major risk factor for increased mortality and poor clinical outcomes in patients with cancer [7]. Chemoresistance can be caused by various mechanisms, including abnormal drug efflux and metabolism, inactivation of cell death and apoptosis, abnormal activation of cell survival-related signaling routes, and enhanced DNA repair mechanisms [8]. In fact, chemoresistance to most anticancer drugs, such as doxorubicin, vincristine, oxaliplatin, paclitaxel, and bortezomib, leads to poor clinical outcomes and is usually accompanied by cancer relapse [9].

Heat shock transcription factor 1 (HSF1) exerts cellular protective effects through the heat stress response (HSR) and unfolded protein response (UPR) pathways, which are both activated by a variety of stress conditions such as thermal injury, ischemia, and endoplasmic reticulum (ER) stress induced by disrupted proteostasis and oxidative stress [10]. Mechanistically, activated HSF1 upregulates the transcription of HSR- and UPR-related target genes, such as HSPA1A, HSPA1B, and DNAJB1, which are crucial components of the molecular chaperone complex, to maintain protein homeostasis (also called proteostasis) [11]. The activity of HSF1 is regulated by phosphorylation. In particular, the activation of HSF1 through phosphorylation of Ser230 by Ca2+/calmodulin-dependent protein kinase II (CaMKII), Ser320 by protein kinase A (PKA), Thr142 by casein kinase 2 (CK2), Ser419 by polo-like kinase 1 (PLK1), and Ser326 by mitogen-activated protein kinase (MAPK) is known to promote cellular adaptation under proteotoxic stress [12]. Accumulating evidence has demonstrated that the HSF1-regulated proteostatic capacity of cells is closely associated with cancer development, progression, and chemoresistance, leading to poor clinical outcomes in patients with cancer [12]. Thus, inhibition of proteostasis in cancer cells by targeting HSF1 is a promising anticancer therapeutic strategy.

Activation of cellular adaptation mechanisms, such as the UPR, against ER stress is known to promote the plasticity of cancer cells, thus dynamically regulating the metastasis, recurrence, evasion of antitumor immunity, and chemoresistance of tumors [13,14]. ER stress and the UPR are tightly regulated by three ER transmembrane proteins, the inositol-requiring enzyme 1 (IRE1), protein kinase R-like ER kinase (PERK), and activating transcription factor 6 (ATF6), which are UPR sensor proteins [15]. Activation of these ER stress sensor proteins in the tumor microenvironment is characterized by high metabolic demand, hypoxia, oxidative stress, protein synthesis overload, and acidosis, which are known to be involved in oncogenic transformation and reprogramming [14]. For instance, the activated PERK is known to promote chemoresistance, prosurvival capacity, angiogenesis, and lipid and amino acid biosynthesis of cancer cells, supporting tumor growth and progression [16,17,18,19,20]. Accumulating evidence has shown that PERK inhibition effectively decreases tumor growth, metastasis, and chemoresistance, demonstrating PERK as a potential molecular target in anticancer therapeutic strategies [14].

In this study, chemoresistance to the anticancer effects of USP7i was observed in various cancer cell lines. Mechanistically, PERK activates and phosphorylates HSF1, thereby conferring resistance to USP7i-induced cancer cell death. These findings suggest a possible limit to the usage of USP7i and highlight the need for the development of strategies to improve upon current cancer treatments using USP7i P22077 and P5091.

## 2. Results

### 2.1. Heat Stress Response (HSR)- and Unfolded Protein Response (UPR)-Associated Genes Are Increased in Ubiquitin-Specific Protease 7 Inhibitor (USP7i)-Resistant Cancer Cells

**Figure 1:**
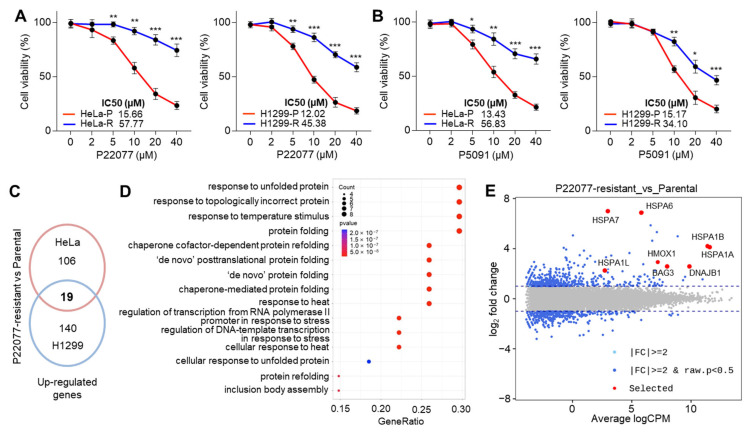
HSR and UPR gene expression in USP7 inhibitor (USP7i)-resistant cancer cells. (**A**,**B**) Cell viability analysis in parental (P) and USP7i-resistant (R) HeLa and H1299 cells in the absence or presence of P22077 (**A**) or P5091 (**B**) for 24 h. Values represent the mean ± SD (n = 4). * *p* < 0.05, ** *p* < 0.0, and *** *p* < 0.001 by one-way ANOVA. Tukey’s post hoc test was performed for statistical analysis. (**C**) Venn diagram showing the 19 common genes upregulated in parental vs P22077-resistant HeLa or H1299 cells. (**D**) Summary of the main GO terms obtained for biological process (BP) in the comparisons of parental vs P22077-resistant HeLa and H1299 cells. (**E**) Smear plot of the log2 fold change (FC) gene expression of P22077-resistant cells compared to parental cells. Blue points represent all upregulated or downregulated genes. Red points represent USP7i-induced HSR- and UPR-associated genes.

Long-term exposure to chemotherapy often leads to the evolution of cancer cells that acquire immune evasion capabilities, resistance to apoptosis, rapid proliferation, and increased invasiveness [21]. Multiple types of ubiquitin-specific protease 7 inhibitors (USP7i) have been discovered and evaluated in preclinical settings as potential anticancer drugs [5,6]. However, the resistance of cancer cells to USP7i has not been evaluated. To this end, we initially tested whether cancer cells show decreased cytotoxicity upon long-term exposure to USP7i and accordingly obtained HeLa and H1299 cells resistant to P22077 and P5091. Compared with parental, these resistant cells displayed higher cell viability after treatment with P22077 (Figure 1A) and P5091 (Figure 1B) in a dose-dependent manner. To understand the precise molecular mechanism by which cancer cells acquire resistance to USP7i-induced apoptosis, we analyzed the changes in the transcriptome between parental and USP7i-resistant cells. We found that 19 genes were upregulated in both USP7i-resistant HeLa and H1299 cells (Figure 1C). Gene enrichment analysis and smear plots showed that the expression of a subset of genes related to the unfolded protein response (UPR), chaperone-mediated protein folding, and heat stress response (HSR) was increased in USP7i-resistant HeLa and H1299 cells (Figure 1D,E). These results demonstrated that USP7i-based chemotherapy can lead to chemoresistance.

### 2.2. USP7i Induces the Expression of HSR- and UPR-Associated Genes in Cancer Cells

**Figure 2:**
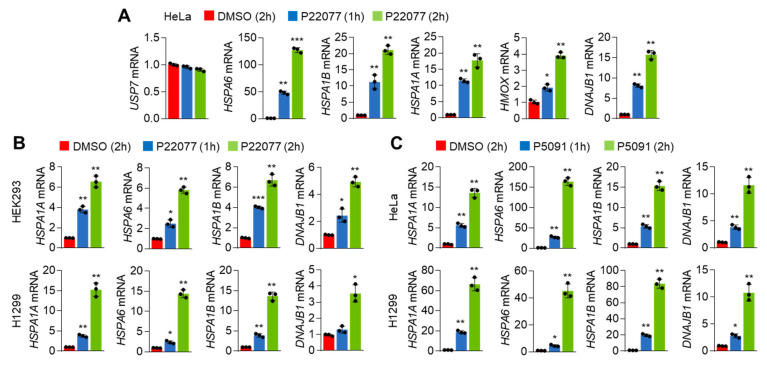
HSR and UPR gene expression in response to USP7i. (**A**,**B**) HSR- and UPR-related gene expression in P22077 (10 µM)-treated HeLa (**A**), HEK293 (**B**, upper), and H1299 (**B**, bottom) cells in a time-dependent manner. (**C**) HSR and UPR gene expression in P5091 (10 µM)-treated HeLa (upper) and H1299 (bottom) cells. The values represent the mean ± SD (n = 3); * *p* < 0.05, ** *p* < 0.01, and *** *p* < 0.001. One-way ANOVA Tukey’s post hoc test was performed for statistical analysis.

Consistent with the RNA-Seq results, we noticed that the expression of HSR- and UPR-associated genes such as HSPA6, HSPA1B, HSPA1A, and DNAJB1 was significantly and rapidly increased in P22077-treated HeLa cells in a time-dependent manner (Figure 2A). We further observed the expression of these genes was also induced in P22077-treated HEK293 and H1299 cells (Figure 2B), suggesting that this mechanism might be common regardless of the cellular context. We also observed that another USP7i, P5091, exerted similar effects on the expression of HSR- and UPR-associated genes in HeLa and H1299 cells (Figure 2C). These results demonstrated that both HSR and UPR are activated in cancer cells in response to USP7i.

### 2.3. HSF1 Is Involved in the Expression of HSR- and UPR-Related Genes in Response to USP7i

**Figure 3:**
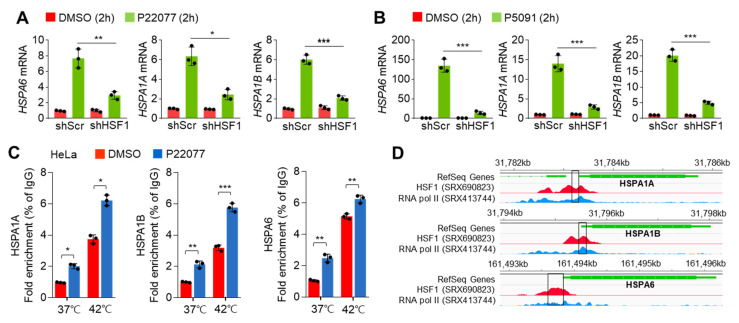
Knockdown effect of HSF1 on USP7i-induced HSR and UPR gene expression. (**A**,**B**) HSR and UPR gene expression in HSF1-silenced HeLa cells in the absence or presence of 10 µM of P22077 (**A**) and 10 µM of P5091 (**B**). (**C**) Promoter occupancy of HSF1 in USP7i-treated HeLa cells under normal (37 °C) or hyperthermia (42 °C) conditions. Cells were pre-incubated with 10 µM of P22077 for 1 h, and the cells were then further incubated under hyperthermia (42 °C) conditions for 1 h. (**D**) Representative images were obtained by ChIP-Atlas (https://chip-atlas.org/, accessed on 10 March 2023) and Integrative Genomics Viewer 2.16.0 (IGV, https://software.broadinstitute.org/software/igv/, accessed on 10 March 2023). Rectangle: PCR amplicon region. Red: HSF1 enriched promoter region, Blue: RNA polymerase II (pol II) enriched promoter region and Green: gene locus and size. The values represent the mean ± SD (n = 3); * *p* < 0.05, ** *p* < 0.01, and *** *p* < 0.001. One-way ANOVA Tukey’s post hoc test was performed for statistical analysis.

HSF1 is a well-known transcription factor that maintains the cellular proteostasis network in response to the HSR in the cytosol and the UPR in the endoplasmic reticulum [22]. Thus, we investigated whether HSF1 is closely related to the induction of the expression of HSR- and UPR-related genes in USP7i-treated cells. To test this, we first generated HSF1-knockdown cells. Figure 3A,B show the expression of HSR- and UPR-related genes was diminished in HSF1-knockdown cells upon P22077 (Figure 3A) and P5091 (Figure 3B) treatment. We then measured the transcriptional activity and target gene promoter-binding activity of HSF1 using chromatin immunoprecipitation (ChIP) to confirm whether HSF1 is functionally active in USP7i-treated cells. We observed the increased promoter binding of HSF1 in HeLa cells exposed to heat shock, used as a positive control (Figure 3C). In addition, we detected a higher occupancy of HSF1 on the promoter regions of HSPA1A, HSPA1B, and HSPA6 genes in cells exposed to both normal temperature and heat shock (Figure 3C). Using the ChIP-Atlas and Integrative Genomics Viewer, we obtained specific nucleotide sequences of the active promoter regions of HSPA1A, HSPA1B, and HSPA6 genes that displayed higher RNA pol II enrichment with HSF1 (Figure 3D). These results revealed that HSF1 is required for the USP7i-induced expression of HSR- and UPR-related genes.

### 2.4. USP7i Leads to HSF1 Phosphorylation

**Figure 4:**
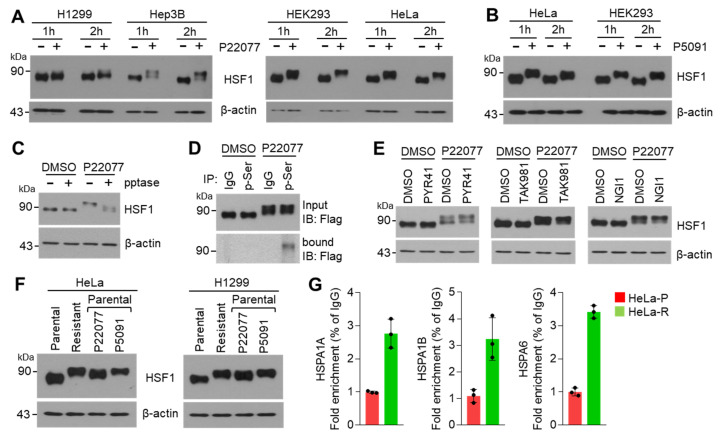
HSF1 phosphorylation by USP7i. (**A**,**B**) A shift in the molecular weight of HSF1 in P22077 (**A**) or P5091 (**B**)-treated cells. H1299, Hep3B, HEK293. and HeLa cells were incubated with 10 µM of P22077 or P5091 for 1 h or 2 h. (**C**) The effect of lambda protein phosphatase (pptase) in USP7i induced a shift in the molecular weight of HSF1. HeLa cells were incubated with 10 µM of P22077 for 2 h, and then total cell lysates were incubated in the absence or presence of pptase for 1 h at 37 °C. (**D**) HEK293 cells were transiently transfected with full-length Flag-HSF1, and cells were then incubated with DMSO or 10 µM of P22077 for 2 h. Phosphorylated HSF1 was measured using immunoprecipitation and Western blotting with indicated antibodies. (**E**) HeLa cells were incubated with inhibitors targeting ubiquitin-activating enzyme E1 (PYR41, 10 µM), sumoylation (TAK981, 1 µM), and glycosylation (NGI1, 10 µM) for 1 h, and the cells were then further incubated in the absence or presence of 10 µM of P22077 for 2 h. (**F**) A shift in the molecular weight of HSF1 in parental or P22077-resistant cells. Parental cells were incubated with 10 µM of P22055 or 10 µM of P5091 for 1 h. (**G**) Occupancy of HSF1 on the promoter region of *HSPA1A*, *HSPA1B,* and *HSPA6* in parental (HeLa-P) or P22077-resistant (HeLa-R) cells.

To understand the precise mechanism by which USP7i activates HSF1, resulting in the induction of expression of HSR- and UPR-related genes, we measured the levels of the HSF1 protein in multiple types of cancer cell lines in the absence or presence of P22077. Surprisingly, we detected higher molecular weight forms of HSF1 in P22077-treated H1299, Hep3B, HEK293, and HeLa cells (Figure 4A). We observed a clear shift in the molecular weight of HSF1 in P5091-treated HEK293 and HeLa cells (Figure 4B), suggesting that HSF1 can be phosphorylated [23,24,25] in response to USP7i. We then tested whether the USP7i-induced shift in the molecular weight of HSF1 was reverted following phosphatase treatment. Western blotting analysis showed the conversion of the upper band of HSF1 into the lower band upon lambda protein phosphatase (pptase) treatment, indicating that this upper band, which appeared after exposure to USP7i, corresponds to the phosphorylated form of HSF1 (Figure 4C). Indeed, an immunoprecipitation (IP) assay using antibodies against phosphorylated serine revealed that P22077 increased the levels of phosphorylated-HSF1 (Figure 4D). Figure 4E reveals that ubiquitination, sumoylation, and glycosylation are not involved in a shift in the molecular weight of HSF1 in response to USP7i. To discover whether phosphorylation and activation of HSF1 is increased in USP7i-resistant cells compared with parental cells, a shift in the molecular weight and transcriptional activity of HSF1 were measured in parental and USP7i-resistant HeLa and H1299 cells. Surprisingly, an increased higher molecular weight of HSF1 was observed in P22077-resistant HeLa and H1299 cells as well as acute exposure of USP7i in parental cells (Figure 4F). Consistently, increased promoter occupancy of HSF1 was found in P22077-resistant HeLa (HeLa-R) cells (Figure 4G). These results indicate that the USP7i induces HSF1 phosphorylation and transcriptional activation.

### 2.5. C-Terminal Domain of HSF1 Is Phosphorylated in Response to USP7i

**Figure 5:**
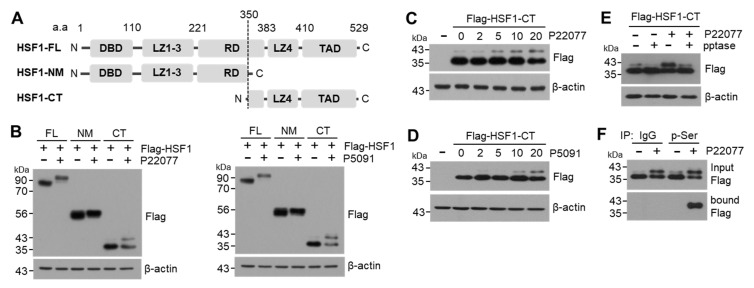
C-terminal domain of HSF1 phosphorylation by USP7i. (**A**) HSF1 full-length (FL, aa 1–529), N-terminal and middle domain of HSF1 (NM, aa 1–350) and C-terminal domain of HSF1 (CT, aa 350–529) used in this study. DBD (DNA-binding domain), LZ (leucine zipper domain), RD (regulatory domain), and TAD (transactivation domain). (**B**) A shift in the molecular weight of HSF1 and mutants. HeLa cells were transiently transfected with Flag-HSF1-FL, -NM, and -CT, and the cells were then incubated with 10 µM of P22077 or P5091 for 2 h. (**C**,**D**) HeLa cells expressing Flag-HSF1-CT were incubated with P22077 (**C**) or P5091 (**D**) for 2 h in a dose-dependent manner. (**E**) HeLa cells expressing Flag-HSF1-CT were incubated with 10 µM of P22077 for 2 h, and then total cell lysates were incubated in the absence or presence of pptase for 1 h at 37 °C. (**F**) HEK293 cells expressing Flag-HSF1-CT were incubated in the absence or presence of P22077 (10 µM) for 2 h. Phosphorylated HSF1-CT was measured using immunoprecipitation and Western blotting with indicated antibodies.

HSF1 is phosphorylated at multiple serine and threonine residues, particularly in the regulatory domain (RD) [26]. To identify the specific domain phosphorylated in response to USP7i, we tested the shift in the molecular weight of HSF1 using mammalian expression vectors harboring HSF1-NM containing the N-terminal and middle domain of HSF1 (aa 1–350) and HSF1-CT containing the C-terminal domain of HSF1 (aa 350–529) (Figure 5A). We observed that the upper band (phosphorylated HSF1) was induced by treatment with USP7i P22077 and P5091 in cells transfected with both HSF1-FL (full-length) and HSF1-CT, whereas it was not detected in cells transfected with HSF1-NM (Figure 5B), indicating that the USP7i-induced phosphorylation of HSF1 occurs in the C-terminal domain containing the leucine zipper domain (LZ, also described as the hydrophobic repeat region) and transactivation domain (TAD). We detected higher molecular weights of HSF1-CT in P22077- (Figure 5C) and P5091-treated (Figure 5D) HeLa cells in a dose-dependent manner. Moreover, we found that the P22077-induced increase in the level of phosphorylated HSF1-CT was significantly reduced by phosphatase treatment (Figure 5E). An IP assay using antibodies against phosphorylated serine showed that P22077 increased the phosphorylation of HSF1 at the C-terminal domain (Figure 5F). Collectively, these results demonstrated that HSF1 phosphorylation occurs in the C-terminal domain that contains the TAD in response to USP7i.

### 2.6. PERK Is Involved in the USP7i-Mediated HSF1 Phosphorylation

**Figure 6:**
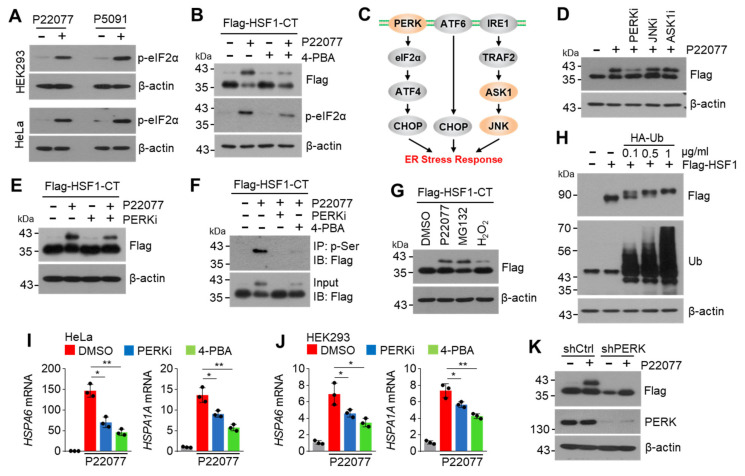
ER stress PERK pathway involves HSF1 phosphorylation and activation. (**A**) Phosphorylated-eIF2α (p-eIF2α), as an ER stress marker, was measured in P22077 (10 µM)- or P5091 (10 µM)-treated cells. (**B**) HEK293 cells transfected with Flag-HSF1-CT were pre-incubated with 2 mM of 4-PBA for 1 h, and the cells were then further incubated with 10 µM of P22077 for 2 h. (**C**) Schematic diagram of signal transduction pathway and key kinases regulating ER stress response. (**D**) Flag-HSF1-CT transfected HEK293 cells were pre-incubated with inhibitors targeting PERK (PERKi, GSK2606414, 100 nM), JNK (JNKi, SP600125, 10 µM), and ASK1 (ASK1i, GS-4997, 20 µM) for 1 h, and the cells were then further incubated with 10 µM of P22077 for 2 h. (**E**) HeLa cells expressing Flag-HSF1-CT were incubated with P22077 (10 µM) and PERKi (100 nM) for 2 h. (**F**) HeLa cells expressing Flag-HSF1-CT were incubated in the absence or presence of P22077 (10 µM), PERKi (100 nM), and 4-PBA (2 mM) for 2 h, as indicated. Phosphorylated HSF1-CT was measured using immunoprecipitation and Western blotting with indicated antibodies. (**G**) HeLa cells expressing Flag-HSF1-CT were incubated in the absence or presence of P22077 (10 µM), MG132 (20 µM), and H_2_O_2_ (50 µM) for 2 h. (**H**) Various concentrations of HA-ubiquitin (Ub) and Flag-HSF1-FL were co-transfected in HEK293 cells. (**I**,**J**) HeLa (**I**) and HEK293 (**J**) cells were pre-incubated with PERKi (100 nM) or 4-PBA (2 mM) for 1 h, and the cells were then further incubated with P22077 (10 µM) for 2 h. (**K**) A shift in the molecular weight of HSF1-CT in control or PERK-silenced HeLa cells. The values represent the mean ± SD (n = 3); * *p* < 0.05 and ** *p* < 0.01. One-way ANOVA Tukey’s post hoc test was performed for statistical analysis.

Previous studies have shown that USP7i lead to endoplasmic reticulum (ER) stress [27]. ER stress-mediated UPR, caused by the accumulation of misfolded proteins, activates HSF1 and its target chaperone proteins, such as HSPA1A, HSPA6, DNAJB1, and HSPA8, to maintain intracellular proteostasis [28]. Therefore, we investigated whether ER stress mediates the USP7i-induced phosphorylation of HSF1. Consistent with previous observations [27], we found that the level of phosphorylated eIF2α, an ER stress marker, was dramatically increased in USP7i-treated HEK293 and HeLa cells (Figure 6A). We also noticed that the P22077-induced increased levels of phosphorylated HSF1-CT and phosphorylated eIF2α were diminished in 4-PBA (an ER stress inhibitor)-treated cells (Figure 6B). Protein kinase R (PKR)-like endoplasmic reticulum kinase (PERK), apoptosis signal-regulating kinase 1 (ASK1), and c-Jun N-terminal kinase (JNK) signaling proteins are involved in cellular adaptation to ER stress, and inhibitors targeting these signaling proteins are known to block the ER stress response (Figure 6C). To discover the kinase that is involved in the phosphorylation of HSF1 in response to USP7i-induced ER stress, we tested the suppressive effect of small molecule inhibitors targeting PERK, ASK1, and JNK on the phosphorylation of HSF1 in ectopic Flag-HSF1-CT-transfected HEK293 cells. We observed that the USP7i-induced increase in the level of phosphorylated HSF1 was decreased in PERK inhibitor (PERKi, GSK2606414)-, but not in JNK inhibitor (JNKi, SP600125)- or ASK1 inhibitor (ASK1i, GS-4997)-treated cells, indicating that PERK is a potential upstream kinase that regulates the phosphorylation of HSF1 (Figure 6D,E). To determine whether ER stress and its sensor PERK are involved in USP7i-induced phosphorylation of HSF1, we measured the level of phosphorylated HSF1-CT in the absence or presence of a PERKi or an ER stress inhibitor (4-PBA). We found that the P22077-induced phosphorylation of serine residues in the C-terminal domain of HSF1 completely disappeared in PERKi- and ER stress inhibitor-treated cells (Figure 6F). Because proteasome inhibitors such as MG132 and oxidative stress have been found to disrupt the UPR and increase ER stress [29,30], we measured the level of phosphorylated HSF1-CT in the absence or presence of MG132 in H2O2-treated cells. Similar to USP7i, we observed the presence of phosphorylated HSF1-CT in the MG132- and H2O2-treated cells (Figure 6G). Consistently, we observed that increased levels of polyubiquitinated proteins, due to the ectopic overexpression of ubiquitin, led to the phosphorylation of HSF1-CT, as indicated by a shift in its molecular weight (Figure 6H). Conversely, administration of PERKi and an ER stress inhibitor (4-PBA) attenuated the P22077-induced expression of HSF1 target HSR- and UPR-related genes, such as HSPA6 and HSPA1A, in HeLa (Figure 6I) and HEK293 (Figure 6J) cells. In addition, we found that PERK knockdown sufficiently blocks a P22077-induced shift in the molecular weight of HSF1-CT in HeLa cells (Figure 6K). These results indicated that PERK, which is activated in response to ER stress, regulates the USP7i-induced phosphorylation of HSF1.

### 2.7. Suppression of the PERK–HSF1 Axis Sensitizes Cells to the Anticancer Effect of USP7i

**Figure 7:**
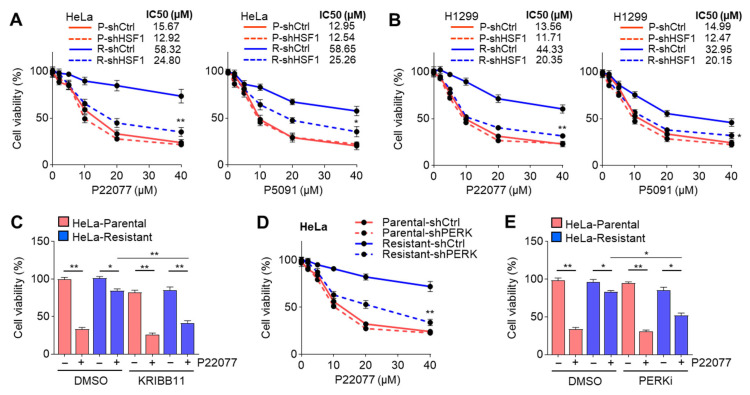
Suppression of HSF1 and PERK sensitizes USP7i-induced cytotoxicity. (**A**,**B**) Cell viability in control (shCtrl) or HSF1 (shHSF1)-silenced parental (P) and USP7i-resistant (R) HeLa (**A**) and H1299 (**B**) cells. Cells were incubated with P22077 and P5091 for 24 h in a dose-dependent manner (2, 5, 10, 20, and 40 µM) as indicated. (**C**) Parental and USP7i-resistant HeLa cells were pre-incubated with KRIBB11 (2 µM) for 2 h, and the cells were then further incubated with P22077 (10 µM) for 24 h. (**D**) Cell viability in control or PERK silenced parental and USP7i-resistant HeLa cells. (**E**) Parental and USP7i-resistant HeLa cells were pre-incubated with PERKi (100 nM) for 2 h, and cells were then further incubated with P22077 (10 µM) for 24 h. Values represent the mean ± SD (n = 4); * *p* < 0.05 and ** *p* < 0.01. One-way ANOVA Tukey’s post hoc test was performed for statistical analysis.

Given that the expression of HSF1 and its target genes participating in HSR is increased in USP7i-resistant cancer cells, we hypothesized that the USP7i-induced phosphorylation and activation of HSF1 lead to resistance to USP7i-based chemotherapy. As expected, HSF1-knockdown largely decreased the viability of both USP7i-treated HeLa (Figure 7A) and H1299 (Figure 7B) cells. Similarly, we observed the increased cytotoxicity of P22077 in HeLa cells treated with the HSF1 inhibitor (KRIBB11) (Figure 7C). Because HSF1 is phosphorylated and activated by PERK in response to ER stress, we next tested whether the inhibition of PERK increases the cytotoxicity of USP7i in a manner similar to the inhibition of HSF1. We found that USP7i-induced cytotoxicity was significantly increased in PERK-silenced (Figure 7D) and PERKi-treated (Figure 7E) HeLa cells. These results indicated that the molecular network of the PERK-mediated phosphorylation and activation of HSF1 is a potential target for overcoming resistance to USP7i-based chemotherapy.

## 3. Discussion

Ubiquitin-specific protease 7 (USP7, also known as HAUSP) is frequently overexpressed in various tumors, including breast, prostate, colorectal, and lung, demonstrating that USP7 is closely associated with tumor development and progression [1]. Numerous preclinical studies have demonstrated the anticancer effects of USP7 inhibitors (USP7i) in multiple types of cancer using in vitro and in vivo models [5]. Various chemical anticancer drugs occasionally display fewer anticancer effects in both experimental animal models and humans because of acquired chemoresistance. For instance, the proteasome inhibitor bortezomib (also known as Velcade, formerly PS-341) is a well-known Food and Drug Administration (FDA)-approved chemical anticancer drug widely used to treat patients with multiple myeloma and mantle cell lymphoma [31]. Although bortezomib has clearly shown an improvement in the 5-year survival rate in patients, many studies have claimed that the anticancer effects of bortezomib gradually decrease due to the upregulation of multidrug resistance (MDR), including pro-survival signaling pathways [32,33]. Mechanistically, upregulation of the expression of heat stress response (HSR)-related genes, such as heat shock protein family A (Hsp70) member 1B (HSPA1B), Hsp70 member 1A (HSPA1A), and DnaJ heat shock protein family (Hsp40) member B1 (DNAJB1), in response to bortezomib was reported to decrease the proapoptotic effects of bortezomib in multiple myeloma cells [34]. Similarly, our data showed that cancer cells show decreased sensitivity to USP7i (P22077 and P5091)-induced cell death. USP7i significantly upregulated HSR by inducing the phosphorylation and transcriptional activity of HSF1. Thus, we speculated that the activation of HSF1 is required for drug resistance against USP7i, such as bortezomib.

This study is the first to demonstrate that phosphorylation of HSF1 confers resistance to cancer cells against USP7i-based chemotherapy. Our unbiased transcriptome and biochemical analysis showed that HSF1 and its target genes associated with HSR and the unfolded protein response (UPR) were upregulated in USP7i-treated cancer cells. Similar associations between nuclear HSF1 and drug resistance against doxorubicin, paclitaxel, carboplatin, and bortezomib have been previously shown in multiple types of cancer cells, such as breast cancer, hepatocellular carcinoma, melanoma, and multiple myeloma cells [10,35,36,37]. Indeed, HSF1-knockdown cells displayed vulnerability to USP7i-induced cell death compared with parental cells. These results suggested that HSF1 is a major contributor to drug resistance against USP7i, supporting that HSF1 is a promising molecular target for overcoming cancer resistance to chemotherapy and providing a plausible strategy for improving chemotherapy efficacy.

HSF1 is ubiquitously expressed in most cell types and tissues, and its transcriptional activity is tightly regulated by post-translational modifications (PTM), such as sumoylation, acetylation, and phosphorylation [38]. The mitogen-activated protein kinase (MAPK)-activated protein kinase 2 (MAPKAPK2)-mediated phosphorylation of the DNA-binding domain (DBD) of HSF1 at serine 121 is known to suppress the transcriptional activity of HSF1 by promoting the physical interaction between HSF1 and its negative regulator, Hsp90 [39]. The regulatory domain (RD), which acts as a repressor of the activity of HSF1 and also as a stress sensor, is phosphorylated at multiple serine residues, such as 230, 292, 303, 307, 326, 344, 363, and 369, by glycogen synthase kinase 3 (GSK3), MAPK, p38 MAPK, mitogen-activated protein kinase (MAPKK, also known as MEK), mammalian target of rapamycin (mTOR), and c-Jun amino-terminal kinase (JNK) [40,41,42]. In particular, JNK phosphorylates HSF1 in the RD and transactivation domain (TAD), consequently activating the HSF1-mediated heat shock response [43]. Likewise, phosphorylation of TAD at serine 419 by polo-like kinase 1 (PLK1) promotes tumorigenesis via TRRAP-TIP60 acetyltransferase-mediated chromatin remodeling [23]. ER stress was also reported to lead to the activation of JNK signaling and its downstream biological processes for cellular adaptation [44]. Our study revealed that JNK was not involved in the phosphorylation of HSF1, as evidenced by the failed reversal of the USP7i-induced phosphorylation of HSF1 by a JNK inhibitor (SP600125). In addition, the phosphorylated form of HSF1-NM (N-terminal and middle domain, aa 1–350) containing DBD and RD was not observed in USP7i-treated cells, indicating that USP7i-mediated phosphorylation of HSF1 occurs in the C-terminal domain containing TAD. Indeed, the phosphorylated form of HSF1-CT (C-terminal domain, aa 350–529) was clearly observed in USP7i-treated cells. Although we succeeded in finding the specific domain of HSF1 that is phosphorylated in response to USP7i and ER stress in this study, further investigation is necessary to identify the specific kinase that directly phosphorylates HSF1 and the specific serine residues phosphorylated in response to ER stress.

The key findings of this study are that a selective protein kinase R (PKR)-like endoplasmic reticulum kinase (PERK) inhibitor, but not a mitogen-activated protein kinase 5 (ASK1, also known as MAP3K5) inhibitor, dramatically reduced the USP7i-induced phosphorylation of HSF1, indicating that PERK acts as a potential upstream kinase of HSF1 for maintaining cellular adaptation to various stress conditions, including cancer cell death caused by chemotherapy. Given that PERK promotes cancer development, growth, survival, and evasion from antitumor immunity, it is plausible that PERK-induced phosphorylation and activation of HSF1 are associated with chemoresistance [45,46]. In fact, our study showed that inhibiting PERK made cancer cells more vulnerable to USP7i-induced cell death. Similar findings have been reported for the forkhead box O3 (FOXO3)-mediated phosphorylation and activation of PERK, which lead to drug resistance to epirubicin and tamoxifen in breast cancer cells [47]. The clinical impact of PERK as a molecular target for overcoming chemoresistance to epirubicin- or tamoxifen-based regimens for treating patients with breast cancer has been previously evaluated; GSK2606414, a PERK inhibitor, was shown to sensitize MCF-7 breast cancer cells to epirubicin and tamoxifen [47]. Additionally, the activation of PERK in response to ER stress was reported to induce the expression of MDR-related protein 1 (MRP1) via nuclear factor erythroid 2-related factor 2 (NRF2), consequently promoting the resistance of HT29 colorectal cancer cells to oxaliplatin [48]. In addition, ER stress was associated with chemoresistance against USP7i, based on data from using 4-PBA, an ER stress inhibitor, which clearly attenuated the USP7i-induced phosphorylation of HSF1 and expression of target HSR-related genes. ER stress partly provides cancer cells the ability to avoid proapoptotic signaling by chemical anticancer drugs [49]. For instance, bortezomib-induced ER stress due to disrupted proteostasis, caused by the accumulation of intracellular polyubiquitinated proteins, has been previously reported [50]. Similarly, Lee et al. previously showed that ER stress and polyubiquitinated proteins were highly increased in both USP7i-treated HCT116 p53+/+ and p53−/− cells [27]. Our data revealed that ER stress-associated PERK contributes to HSF1-mediated chemoresistance against USP7i. Therefore, our observations support that ER stress and PERK signaling are potential targets for overcoming chemoresistance to proteotoxicity-inducing chemical anticancer drugs, such as bortezomib and USP7i.

In conclusion, the major findings were that: (1) following prolonged exposure, cells become insensitive to USP7i-induced cell death; (2) ER stress-associated PERK is required for the activation and phosphorylation of HSF1 in USP7i-chemoresistant cells; and (3) suppression of HSF1 and PERK sensitizes cells to the anticancer effects of USP7i. Overall, the present study provided a possible therapeutic strategy based on the combined treatment with USP7i and HSF1- or PERK-targeting drugs for effectively killing cancer cells.

## 4. Materials and Methods

### 4.1. RNA-Sequencing and Quantitative Real-Time PCR (qRT-PCR)

TRIzol (Invitrogen, Carlsbad, CA, USA) and isopropanol (Sigma-Aldrich, St. Louis, MO, USA) were used to isolate total RNA in cultured cells. An amount of 1 × 10^6^ cells were lysed by using 1 mL of TRIzol. Total RNA was precipitated by using isopropanol and washed using 80% precooled ethanol. RNA purity was measured by using a spectrophotometer (BioTek, Winooski, VT, USA) at 260 and 280 nm, and a ratio of ~1.8 was accepted for qRT-PCR. A reverse transcriptase and cDNA synthesis kit (Applied Biosystems, Foster City, CA, USA) were used for cDNA synthesis. SYBR Green qPCR mixture (Applied Biosystems, Foster City, CA, USA) was used for PCR amplification and was run for 40 cycles using a LightCycler 96 System (Roche Diagnostics K.K., Tokyo, Japan). The 2^−∆∆Ct^ method using human 36B4 (ribosomal protein subunit P0, RPLP0) as a reference gene was used for calculating gene expression. Detailed primer sequences for qRT-PCR are shown in Table 1.

For bulk RNA-Seq, the RNA was isolated from both parental and P22077-resistant HeLa and H1299 cells by using TRIzol (Invitrogen, Carlsbad, CA, USA). The RNA-Seq library preparation was performed by Macrogen Co., Ltd., (Seoul, Republic of Korea) using a TruSeq Stranded mRNA LT Sample Prep Kit (Illumina, Inc., Hayward, CA, USA) after quality control and was subsequently sequenced on a NovaSeq 6000 System (Illumina, Inc., Hayward, CA, USA). Trimmomatic v0.39 was used for adaptor removal and quality trimming, and trimmed and corrected RNA-Seq reads were aligned to the human reference genome by using HISAT2 v2.1.0. StringTie v1.3.4 was used to calculate and estimate transcript expression levels. The read count data were processed based on the quantile normalization method using EdgeR within R (R Development Core Team, 2016) using Bioconductor [51]. Differentially expressed genes (DEGs) between comparison samples were determined using EdgeR (exactTest). Fold change (FC) and CPM (counts per million reads) values were used for visualization of gene expression data. Gene Ontology (GO) enrichment and functional annotation analysis for the up- and downregulated DEGs were performed using gProfileR (https://biit.cs.ut.ee/gprofiler/, accessed on 1 September 2022).

### 4.2. Co-Immunoprecipitation and Western Blotting

To detect phosphorylated HSF1, HEK293 cells were transiently transfected with Flag-HSF1-FL (full-length) and Flag-HSF1-CT (C-terminal domain), respectively, and then transfected HEK293 cells were further incubated in the absence or presence of P22077. Total cell lysates were obtained by using a lysis buffer containing 1% NP-40, 150 mM NaCl, 50 mM Tris-HCl (pH 7.9), 0.1 mM ethylenediaminetetraacetic acid (EDTA), and a protease inhibitor cocktail. Cell lysates (2 mg/mL) were incubated with 1 μg of antibody-recognizing phosphorylated-serine for 16 h at 4°C, and then the immune complexes were gently washed with wash buffer containing 0.5% NP-40, 200 mM of NaCl, 50 mM of Tris-HCl (pH 7.9), 0.1 mM of EDTA, and protease inhibitor cocktail. The immunoprecipitated protein complex was dissolved with SDS (sodium dodecyl sulfate) sample buffer (50 mM of Tris-HCl pH6.8, 2% SDS, 10% glycerol, 1% β-mercaptoethanol, and 12.5 mM of EDTA, 0.02% bromophenol blue). For Western blotting, total protein was extracted from the cells with NP-40 lysis buffer containing 1% NP-40, 2.5M of NaCl, 1M of Tris-HCl, 500 Mm of EDTA, 1 M of NaF, and protease inhibitor cocktail. A Bradford protein assay was conducted to determine total protein concentration. Total proteins were resolved by SDS (sodium dodecyl sulfate)-PAGE (polyacrylamide gel electrophoresis), then transferred onto PVDF membranes (Millipore, Burlington, MA, USA) and blocked with 5% skimmed milk (*w*/*v*) in TBST buffer incubated with the indicated primary antibodies (1:1000) overnight at 4 °C. The membranes were further incubated with the corresponding HRP-conjugated secondary antibodies (1:10,000, Jackson ImmunoResearch Laboratories, West Grove, PA, USA) for 1 h at room temperature. Antibodies against phosphorylated serine (Cat# 612546), β-actin (Cat# sc-47778), Flag-tag (Cat# F3165), p-eIF2α (Cat# 9721), HSF1 (Cat# 12972), and ubiquitin (Cat# sc-8017) were purchased from Santa Cruz Biotechnology (Santa Cruz, CA, USA), Sigma-Aldrich (St. Louis, MO, USA), and Cell Signaling Technology (Danvers, MA, USA). Finally, protein expression was visualized using an ECL Prime kit (GE Healthcare, Milwaukee, WI, USA).

### 4.3. Cell Viability Assay

Crystal violet (Sigma-Aldrich, St. Louis, MO, USA) was used to measure cell viability [52]. Cells were seeded at 2 × 10^5^ cells/well in 24-well tissue culture plates and incubated for 24 h in the absence or presence of P22077 or P5091. After drug treatment, the cells were washed three times using cold phosphate-buffered saline (PBS) and fixed with 4% paraformaldehyde, and then the fixed cells were incubated with 0.5 mL of crystal violet solution for 20 min at room temperature. The optical density (OD) of the number of live cells was measured at 570 nm by using an absorbance reader (BioTek, Winooski, VT, USA).

### 4.4. Cell Culture, Drug Treatment, and Chemical Reagents

HeLa (10002), H1299 (25803), HEK293 (21573), and Hep3B (88064) cancer cells were obtained from Korean Cell Line Bank (Seoul, Republic of Korea). Minimum essential medium (MEM), Roswell Park Memorial Institute (RPMI) 1640, and Dulbecco’s modified Eagle’s medium (DMEM) containing 10% fetal bovine serum (FBS) and antibiotics were used for cell culture, respectively. The USP7i-resistant cell lines HeLa (HeLa-P) and H1299 (H1299-P) were established by exposing each cell line to increasing concentrations at 1 µM to 20 µM of P22077 or P5091. In brief, the cells were incubated to low doses of P22077 or P5091 starting at 1 µM. At every two subcultures at 70% confluence, the concentration of P22077 or P5091 was gradually increased until it reached 20 µM. The establishment of a stable P22077- or P5091-resistant subline was determined by comparing the IC50 values of parental and resistant cells after growing them in a P22077- or P5091-free culture medium for at least 2 weeks. P22077 (10 µM) and P5091 (10 µM) were treated into HeLa, H1299, and HEK293 cells for 1 h or 2 h, respectively, before measuring molecular weight shift and phosphorylation of HSF1, and heat stress response (HSR)- and unfolded protein response (UPR)-related gene expression. P22077 (Cat# S7133), P5091 (Cat# S7132), ASK1 inhibitor (GS-4997, Cat# S8292), PYR41 (Cat# S7129), TAK981 (Cat# S8829), and NGI1 (Cat# S8750) were purchased from Selleckchem (Houston, TX, USA). KRIBB11 (Cat# 385570) was purchased from Santa Cruz Biotechnology (Santa Cruz, CA, USA). Lambda protein phosphatase (Cat# P9614), PERK inhibitor (GSK2606414, Cat# 516535), JNK inhibitor (SP600125, Cat# 420119), hydrogen peroxide (H2O2, Cat# H1009), 4-Phenylbutyric acid (4-PBA, Cat# P21005), and MG132 (Cat# M7449) were purchased from Sigma-Aldrich (St. Louis, MO, USA).

### 4.5. Plasmids, Transfection, and Lentiviral Transduction

Transient transfection was performed using Polyfect transfection reagent (Qiagen, Valencia, CA, USA). HEK293T cells were seeded at 0.3 × 10^6^ cells/well in 6-well plates, and the cells were then transiently transfected with Flag-HSF1. After transfection, the cells were incubated for 48 h to allow expression and accumulation of target proteins, and the cells were then used to measure molecular weight shift and phosphorylation of HSF1. pLKO.1-shRNA-HSF1 (TRCN0000007482 and TRCN0000007480, Sigma Aldrich) was used to generate HSF1 knockdown cells as described previously [53]. To produce lentivirus-harboring shRNA against HSF1, pLKO.1-shRNA vector, envelope vector (pMD2.G), and packaging vector (psPAX2) were transiently transfected into the HEK293T cells using Polyfect reagent (Qiagen, Valencia, CA, USA). The transfected HEK293T cells were incubated for 48 h with 30% FBS-containing culture medium to allow amplification of lentiviruses, and the lentiviral particles were then concentrated and purified by using a Millipore membrane (Burlington, MA, USA) Lentivirus Purification kit [52]. The Flag-HSF1 (Addgene plasmid # 32537) was a gift from Stuart Calderwood [54]. Mammalian expressing Flag-HSF1-NM (aa 1–350) and -CT (aa 350–529) were generated using PCR-containing primers with EcoRI and XhoI restriction sites, and then amplified PCR fragments were inserted into the pcDNA3.1-Flag vector.

### 4.6. Chromatin Immunoprecipitation and Polymerase Chain Reaction (ChIP-PCR)

An EZ-ChIP assay kit (Millipore, Burlington, MA, USA) was used to carry out a chromatin immunoprecipitation (ChIP) assay according to the manufacturer’s instructions and slight modifications as previously described [52]. Briefly, HeLa cells were incubated for 24 h in the absence or presence of P22077, and the cells were then fixed in 1% formaldehyde for 10 min at room temperature. Then, 125 mM of glycine solution was used for the removal of formaldehyde, and then the cells were rinsed twice with cold PBS. The cells were resuspended in ChIP-lysis buffer containing SDS, protease inhibitor cocktail, and PMSF, and lysed using an ultrasonic homogenizer (Bandelin Electronic, Berlin, Germany) for four cycles of 5 minutes (30 seconds on, 30 seconds off, on 30% power) as described previously [52]. Equal amounts of cell lysates were incubated with primary antibodies against normal rabbit serum (IgG) and HSF1 (Cat# 12972, Cell Signaling Technology, Danvers, MA, USA) overnight at 4 °C, and then the immune complexes were recovered using protein A or G agarose beads (Millipore, Burlington, MA, USA) pre-blocked using salmon sperm DNA (Millipore, Burlington, MA, USA). After the primary antibody reaction, the samples were washed three times using wash buffer followed by low salt (0.15 M NaCl), high salt (0.5 M NaCl), lithium chloride (0.25 M LiCl), and Tris-EDTA (TE) buffers, respectively. Immunoprecipitated DNA was purified using a phenol:chloroform:isoamyl alcohol (25:24:1). Quantitative real-time PCR was performed by using SYBR Green Mixture (Applied Biosystems, Waltham, MA, USA) to measure HSF1 enrichment on the promoter regions of HSPA1A, HSPA1B, and HSPA6. DNA enrichment was calculated by normalization using values of normal IgG control. The sequences of ChIP-PCR primers are shown in Table 2.

### 4.7. Statistical Analysis

All the data were analyzed and represented using GraphPad Prism v8.0.1 (GraphPad Software Inc., San Diego, CA, USA). Results are expressed as the mean ± standard deviation (SD). Statistical analysis was carried out using one-way ANOVA with Tukey’s post hoc test for multiple comparisons. A *p*-value < 0.05 was considered statistically significant.

## Figures and Tables

**Table 1:** Primer sequences for qRT-PCR.

Gene	Forward Sequences (5′-3′)	Reverse Sequences (5′-3′)
USP7	CGAGGACATGGAGATGGAAG	GTTGTGTCCATCACTCAGGG
HSPA6	GCGCAAAATGCAAGACAAGTG	GAGAAGATGGGGCGACAGATT
HSPA1B	AGGCCAACAAGATCACCATC	TCGTCCTCCGCTTTGTACTT
HSPA1A	GCCGAGAAGGACGAGTTTGA	TCCGCTGATGATGGGGTTAC
HMOX	AAGATTGCCCAGAAAGCCCTGGAC	AACTGTCGCCACCAGAAAGCTGAG
DNAJB1	GACCCTCATGCCATGTTTGC	CCCATAGGGAAGCCAGAGAAT
β-actin	ACGAGGCCCAGAGCAAGAG	TCTCCAAGTCGTCCCAGTTG

**Table 2:** Primer sequences for ChIP-PCR.

Gene	Forward Sequences (5′-3′)	Reverse Sequences (5′-3′)
HSPA1A	GGCGAAACCCCTGGAATATTCCCGA	AGCCTTGGGACAACGGGAG
HSPA6	GGAAGGTGCGGGAAGGTTCG	TTCTTGTCGGATGCTGGA
HSPA1B	GGTCCGCTTCGTCTTTCG	CTCTGTGGGCTCCGCTCT

## Data Availability

The RNA-Seq data generated in this study are publicly available in the Gene Expression Omnibus (GEO) database under accession GSE213931.
